# Recognition Dynamics of Cancer Mutations on the ERp57-Tapasin Interface

**DOI:** 10.3390/cancers12030737

**Published:** 2020-03-20

**Authors:** Monikaben Padariya, Umesh Kalathiya, Douglas R. Houston, Javier Antonio Alfaro

**Affiliations:** 1 International Centre for Cancer Vaccine Science, University of Gdansk, Wita Stwosza 63, 80-308 Gdansk, Poland; umesh.kalathiya@ug.edu.pl; 2 Institute of Quantitative Biology, Biochemistry and Biotechnology, University of Edinburgh, Edinburgh, Scotland EH9 3BF, UK; douglasr.houston@ed.ac.uk; 3 Institute of Genetics and Molecular Medicine, University of Edinburgh, Edinburgh, Scotland EH4 2XR, UK

**Keywords:** tapasin, ERp57, MHC-I, peptide loading complex, molecular dynamics, stability change, cBioPortal, protein–protein interactions, cancer mutations

## Abstract

Down regulation of the major histocompatibility class (MHC) I pathway plays an important role in tumour development, and can be achieved by suppression of HLA expression or mutations in the MHC peptide-binding pocket. The peptide-loading complex (PLC) loads peptides on the MHC-I molecule in a dynamic multi-step assembly process. The effects of cancer variants on ERp57 and tapasin components from the MHC-I pathway is less known, and they could have an impact on antigen presentation. Applying computational approaches, we analysed whether the ERp57-tapasin binding might be altered by missense mutations. The variants H408R(ERp57) and P96L, D100A, G183R(tapasin) at the protein–protein interface improved protein stability (ΔΔG) during the initial screen of 14 different variants. The H408R(ERp57) and P96L(tapasin) variants, located close to disulphide bonds, were further studied by molecular dynamics (MD). Identifying intramolecular a-a’ domain interactions, MD revealed open and closed conformations of ERp57 in the presence and absence of tapasin. In wild-type and mutant ERp57-tapasin complexes, residues Val97, Ser98, Tyr100, Trp405, Gly407(ERp57) and Asn94, Cys95, Arg97, Asp100(tapasin) formed common H-bond interactions. Moreover, comparing the H-bond networks for P96L and H408R with each other, suggests that P96L(tapasin) improved ERp57-tapasin binding more than the H408R(ERp57) mutant. During MD, the C-terminus domain (that binds MHC-I) in tapasin from the ERp57(H408R)-tapasin complex moved away from the PLC, whereas in the ERp57-tapasin(P96L) system was oppositely displaced. These findings can have implications for the function of PLC and, ultimately, for the presentation of MHC-I peptide complex on the tumour cell surface.

## 1. Introduction

**Figure 1:**
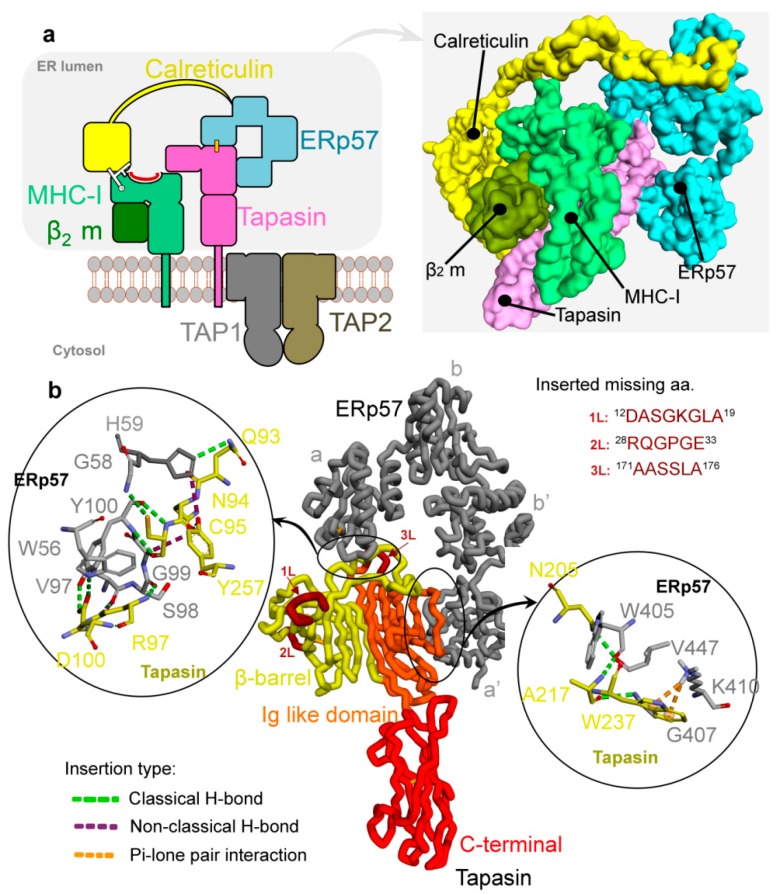
Structures of the peptide-loading complex(PLC) components and ERp57-tapasin complex. (**a**) Schematic representation of individual assembly factors of the PLC showing TAP1, TAP2, tapasin, MHC-I heavy chain (hc), β_2_m, calreticulin, and ERp57 (disulphide bridge is shown as orange line and monoglucosylated (G) N-glycan as white lines) [4], and the structural architecture of fully assembled PLC (PDB ID: 6ENY) [4]. (**b**) The structure and interactions (H-bonds) of the ERp57-tapasin wild-type heterodimer (PDB ID: 3F8U) [22]. N-terminal domain of tapasin is a fusion of β-barrel (residues 1–147) and an Ig-like domain (residues 150-269), and the C-terminus domain (residues range 270–381) of tapasin is similar to the Ig-like domain [22]. Interaction types include classical hydrogen (conventional H-bonds; coloured in green), non-classical hydrogen (carbon hydrogen bond; in purple), and Pi-lone pair interactions (Pi-donor; in orange colour). Missing residues (range: 12–19, 28–33, and 171–176) in the tapasin structure were inserted and shown as dark red colour regions with labels 1L, 2L, and 3L, respectively. Colour scheme: carbon in grey (ERp55) and yellow (tapasin), oxygen in red, nitrogen in blue, hydrogen in silver, and sulphur in orange.

The host immune system plays an important role in the elimination of cancer where the evasion of the immune response is a significant event in tumour development, one of the hallmarks of cancer [1,2]. One of the principal characteristics used by tumour cells is a reduction in immune recognition by targeting antigen processing and presentation, allowing the transformed cells to survive [1]. The cellular path of adaptive immunity employs T-lymphocytes recognizing antigenic peptides presented by major histocompatibility complex (MHC). To be capable of engaging the key components of adaptive immunity, antigens must be processed and presented to immune cells and this process occurs mainly in the peptide-loading complex (PLC) [1,2,3,4,5]. The PLC contains two TAP (TAP1 and TAP2) peptide transporters, delivering antigen peptide substrates into the endoplasmic reticulum (ER), the two chaperones tapasin and calreticulin, and the oxidoreductase ERp57 that, together with MHC-I heterodimer, is required for the efficient loading of antigenic peptides onto MHC-I molecules (Figure 1a) [4,5,6,7,8]. The peptides between 8 and 16 residues long are efficiently translocated by TAP [9,10,11,12], with N-extended precursors transferred more efficiently than mature epitopes [13,14,15,16,17,18]. Nascent MHC-I heavy chains are chaperoned by the calreticulin system in the ER and together with β_2_-microglobulin (β_2_m), MHC-I heavy chains assemble into heterodimers that acts as receptors for antigenic peptides [4,19]. The empty MHC-I heterodimers are recruited by calreticulin and become part of the transient macromolecular PLC [18,20,21], where they are further stabilised by the tapasin protein [4].

A significant fraction of ERp57 (a membrane of protein disulphide isomerase (PDI) family) is covalently associated with the tapasin protein, as shown in Figure 1b [22,23]. In the steady state conditions, about 20% of ERp57 is covalently bound to tapasin in both human and mouse cells, and this fraction increases up to 50–85% in cells activated with interferon-γ [23,24,25,26]. Moreover, in vivo evidence suggests that the functional unit responsible for peptide editing is the covalent disulphide bonded ERp57-tapasin heterodimer [5,27,28]. The stable ERp57-tapasin interaction helps to stabilise other non-covalent interactions between components of the peptide loading complex [29], and it has been shown that the ERp57 protein is indeed a central structural component of the PLC [23] (Figure 1b).

Tapasin forms a L-shaped structure containing the N-terminal (fusion of β-barrel and an Ig-like domain) and the C-terminal (similar to Ig-like domain) domains, whereas the ERp57 is arranged in its typical twisted U-shaped (horseshoe-like) conformation. The tapasin N- and C-terminal domains are both stabilised by disulphide bonds, between Cys7 and Cys71 and between Cys295 and Cys362, respectively [22,29,30]. The ERp57 protein is composed of four (a, b, b’, and a’) thioredoxin-like domains, from which the a and a’ domains have the active-site motifs; C57-G58-H59-C60 and C406-G407-H408-C409, respectively [22,23]. The catalytically active a and a′ domains of ERp57 are complexed to the N-terminal domain of the tapasin protein (Figure 1b). The domain of ERp57 interacts with a long loop in the β-barrel of the tapasin N-terminal domain, and Cys95 residue of this loop forms a covalent disulphide bond with Cys57 of the ERp57 catalytic motif. The a’ domain of ERp57 forms an interface with the Ig-like domain of the tapasin N-terminus [22,29]. It has been shown that the association of tapasin with both active sites of ERp57 has strong implications concerning the role of ERp57 in the PLC [22]. In addition, studies [26,28,31] also support a model in which tapasin has adapted the quality control machinery by covalently sequestering ERp57 to enhance the recruitment and stability within the PLC of empty MHC-I molecules associated with calreticulin, which interacts independently with the class I glycan and the bb’ domains of ERp57 (Figure 1) [22,32].

The ability of a protein to make interactions with macromolecular partners is a critical prerequisite for proper biological function, and a missense mutation affecting protein interactions may lead to significant perturbations of the protein function. The three-dimensional structures of proteins and their complexes could provide important or detailed information to identify cancer-driving mutations [33]. In this work, a subset of cancer-related variants known to be in PDIA3 (ERp57) and TAPBP (tapasin) genes were collected from cBioPortal [34], to access data from the cancer genome atlas (TCGA) and other genomic studies. A collective mutational landscape of ERp57 and tapasin (Figure S1) gives a wide spectrum of variants from different cancer types, and these mutations can have a functional impact. Different cancer types in which the ERp57 and tapasin genes have shown the maximum alteration are endometrial carcinoma, ovarian cancer, melanoma, and ovarian epithelial tumour (Figure S1).

Subsequently, we performed an analysis of intermolecular interactions from the ERp57-tapasin complex in the context of different mutations found associated with cancer at the protein–protein interface. Since, variants from the ERp57-tapasin interface can have a significant effect on the conformational dynamics of the ERp57 and tapasin proteins, and may also have an impact on the PLC. The effects of the variants at the interacting interface were studied using the computational approaches, that represent the wild-type residues structural and chemical environment of a residue as a graph-based signature to determine the change upon mutation in Gibb’s free energy of stability or binding [35]. Variants having considerable effects on ERp57 and tapasin stability or binding were studied extensively by a molecular dynamics (MD) simulations approach. Moreover, in vivo evidence suggests that the covalent ERp57-tapasin heterodimer is the functional unit responsible for the peptide editing [5,27,28], and they are 50–85% covalently bound in cells activated with interferon-γ [23,24,25,26]. Therefore, to understand the effect of covalent bonds on protein–protein interface we simulated model systems in which the Cys57(ERp57)-Cys95(tapasin) are in disulphide bonded and non-bonded state. The molecular dynamics simulations helped to provide molecular insights in elaborating the change in interactions and structural conformations coupled with the cancer associated mutations, and to better understand the implication of the ERp57-tapasin complex in the antigen presentation.

## 2. Results and Discussion

**Figure 2:**
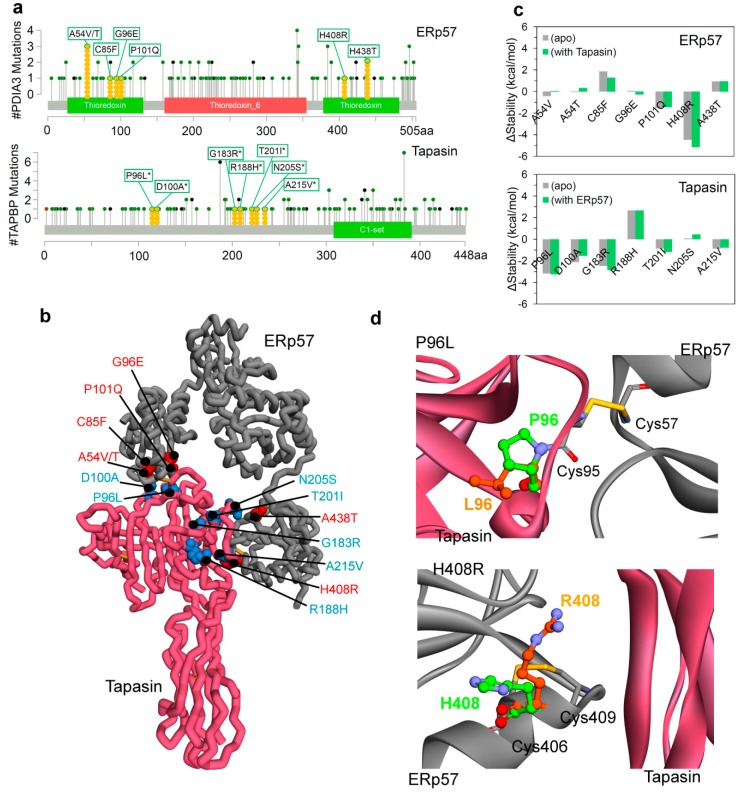
Residues of ERp57 and tapasin proteins that were found mutated in different cancer types. (**a**) Mutation data retrieved from the cBioPortal for PDIA3 (ERp57) and tapasin(TAPBP) genes (* numbering of residues as per the structure obtained from the PDB database for tapasin). (**b**) Cancer mutations located at the protein–protein interface of the ERp57-tapasin complex. (**c**) Changes in thermostability (Δstability or ΔΔG) of the ERp57 and tapasin proteins brought about by mutation. (**d**) Conformational change of the mutant residues (P96L and H408R) obtained during the initial screen of the stability change upon mutation with respect to the native conformation of residues from the X-ray structure. In addition, residues involved in the intermolecular and intramolecular disulphide bonds (Cys57-Cys95 and Cys406-Cys409, respectively) are also shown. Colour scheme: carbon in mutated residues are in orange and native structure are in green, oxygen in red, hydrogen in silver, nitrogen in blue, and sulphur in light orange.

A number of cancer-associated mutations were found in the coding region of PDIA3 and TAPBP genes from the cBioPortal database (the cancer genomics data; Figure 2a and Figure S1) [34]. However, 14 different variants (Figure 2a,b) from the cancer genome atlas (TCGA) sequencing data that reside at the protein–protein or interacting interfaces might have a significant influence on the ERp57-tapasin complex, and therefore, were considered in this work. The findings from in silico mutagenesis analysis identified that H408R mutation in ERp57 improved its structural stability (−5 kcal/mol), and in the case of the tapasin protein mutations P96L, D100A, and G183R improved stability with ΔΔG (Δstability change) values of approximately −3, −2, and −3 (kcal/mol), respectively. In addition, the structural location of each variant suggests that the mutant H408R of ERp57 is placed in the vicinity of the intramolecular disulphide bond (Cys405-Cys409), and the mutant P96L of tapasin is located in the region of the intermolecular disulphide bond between ERp57-tapasin (Cys57-Cys95). The mutant H408R come from the active-site motifs (Cys-Gly-His-Cys) of the a and a’ domains in ERp57, catalytically active motif in a domain is Cys57-Gly58-His59-Cys60 and in a’ domain is Cys406-Gly407-His408-Cys409 [22,36]. Therefore, considering findings from the initial mutation screen and their importance in the active site cleft; mutants H408R(ERp57) and P96L(tapasin) close to the disulphide bonds (functional unit responsible for peptide editing [5,27,28]) were further investigated by molecular dynamics simulations (Figure 2c,d) in order to trace change in the intermolecular (ERp57-tapasin) interactions and dynamics of proteins upon mutation.

### 2.1. Open and Closed Conformation of ERp57, and ERp57-Tapasin Wild-Type Systems

**Figure 3:**
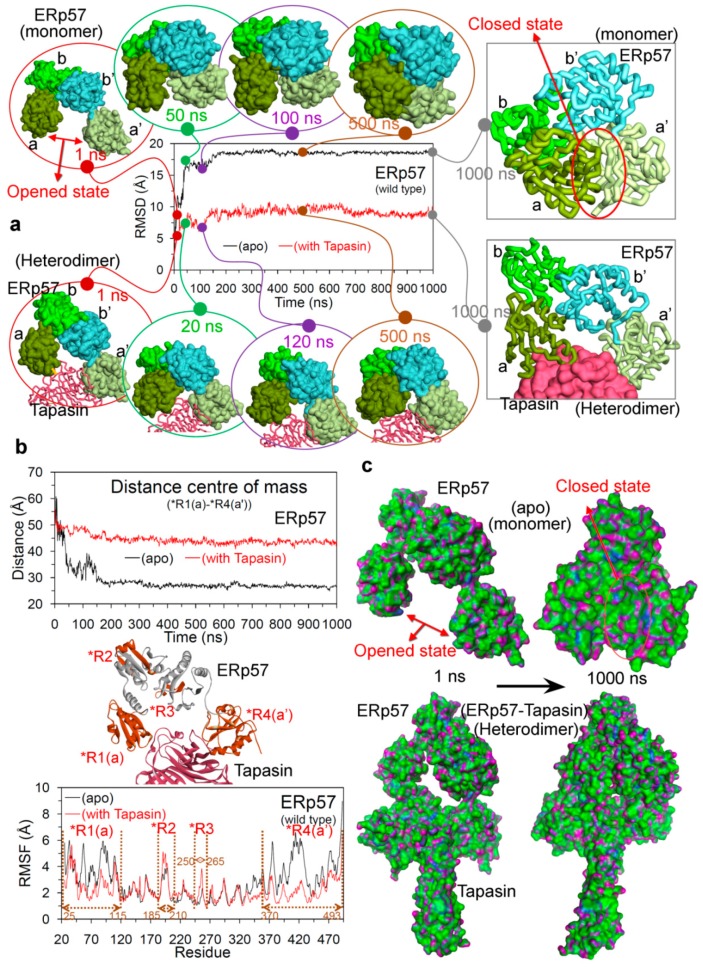
Structural analysis of the wild-type ERp57 from molecular dynamics (MD) simulations. (**a**) Root-mean-square deviation (RMSD) of all-atoms (excluding hydrogen atoms) of ERp57 in apo state and complexed with tapasin. Domain motion modes of ERp57 structure at different time intervals represented the dominant motions of a and a’ domains in the opened and closed conformation/state. (**b**) Distances between the centre of masses of the a and a’ domains of ERp57 and the residual fluctuations (RMSF; root-mean-square fluctuation) of each residue. (**c**) Structure conformations showing the switch of apo ERp57 from open to closed state, and ERp57 in complex with tapasin in its native conformation throughout the 1000 ns MD simulations (colour scheme: green colour for the hydrophobic regions, blue for mildly polar regions (e.g., the hydrogens of benzene) and purple for the hydrogen bonding regions).

To understand the conformational dynamics and change in the protein–protein interactions upon mutations, the MD simulations were performed for 10 different systems (four wild-type systems and six mutant systems). For the wild-type systems, MD simulations of 1000 ns were carried out for the ERp57-tapasin complex (hetero-dimer; PDB: 3F8U [22]) in two forms: bonded through disulphide covalent bond Cys57(ERp57)-Cys95(tapasin) and in the non-bonded form (Figure S2a). Additionally, the ERp57 and tapasin wild-type proteins were also simulated for 1000 ns in their apo state (monomer form; structure retrieved from PDB: 3F8U [22]) to explore their properties when they are alone in the simulation box without their respective partner. The conformational and geometrical properties were analysed from the MD trajectories to understand the structural stability and dynamic changes. The findings from MD suggest that in the beginning of MD simulation (Figure 3a) the ERp57 apo state structure had its native conformation (termed as open conformation in this work) similar as in the X-ray [22], while after ~50 ns the catalytically active a and a’ domains of ERp57 increased their closeness and by the end of MD simulation these domains made interactions with each other (forming a closed conformation for ERp57; Figure 3). For the ERp57 protein, the RMSD (root-mean-square deviation) was found to be relatively a much higher value of ~19 Å in apo state, and that was due to the structural movements by the catalytically active a and a’ domains to form a closed state (Figure 3a). Whereas, in the presence of tapasin in the system (i.e., ERp57-tapasin complex), ERp57 has obtained RMSD values of ~8–9 Å and the binding of tapasin stabilised both a and a’ domains of ERp57 in their native or the open conformation, similar as in the X-ray structure [22] (Figure 3a). Furthermore, it has been observed that RMSD for the ERp57 protein became stable after initial deviation in both monomer and heterodimer systems (apo-form and ERp57-tapasin complex; Figure 3a).

Open and closed conformations of ERp57 relative to the presence and absence of tapasin were also supported by the distance centre of masses obtained between a (*R1) and a’ (*R4) domains. For the apo-form of ERp57 the obtained distance between a and a’ domains was relatively low of ~28 Å, whereas in the presence of tapasin the distance was ~45 Å (Figure 3). These findings from MD correlates with the data from Dong et al. and Tian et al. [22,37] that in the ERp57-tapasin complex, the distance between a and a’ domains measured by the distance between the Ca atoms in Cys57 and Cys406 in ERp57 is ~34 A˚ (open conformation) [22,37], and it is the orientation required for ERp57 to make interaction with tapasin. Considering the open and closed conformation in the ERp57 protein very little is known, whereas the PDI protein from the same disulphide isomerase family is highly studied with crystal structures available in both states (open; oxidised and closed; reduced) [38]. Both ERp57 and PDI are multifunctional proteins with almost similar four domain architectures [38,39,40]. For the PDI protein it is known that the targeted domain rearrangement occurs during redox-dependent activities that leads to open (oxidised) and closed (reduced) conformation [38,41]. In addition, such conformational transition of oxidised/reduced for PDI results in switching its enzymatic activities, and this is under the influence of a’ domain (disulphide bond) leading to the conformational change in the b’ and a’ domains [38,40,42]. Previous studies have traced the redox-dependent opening-closing structure for PDI protein [41] and suggested that a and a’ domain move toward each other increasing the possibility of electron transfer during catalytic activities [43,44]. Similar findings about the movement of a and a’ domain in ERp57 from this work, suggest that such analysis can be helpful to understand the properties of this protein.

**Figure 4:**
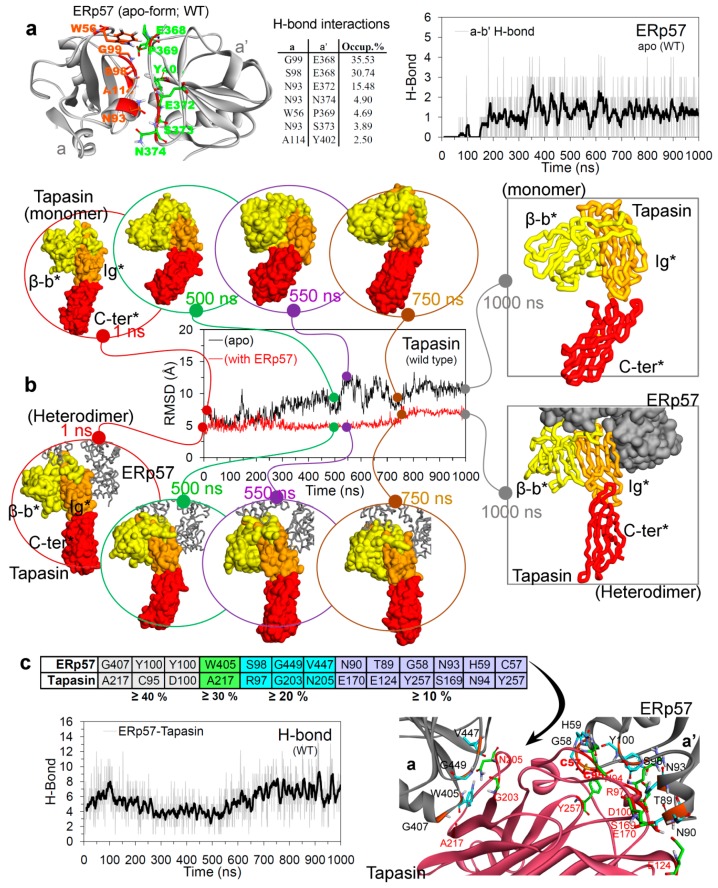
Closed conformation of ERp57 (apo state) and H-bond interaction between ERp57-tapasin. (**a**) Hydrogen bond interactions between the a and a’ domain of wild-type ERp57 in apo state, residues forming interactions with occupancy ≥ 1% are highlighted. Colour scheme: oxygen in red, hydrogen in silver, nitrogen in blue, and sulphur in orange. (**b**) RMSD of all-atoms (excluding hydrogen atoms) of tapasin in apo state and complexed with ERp57. Domain motion modes of tapasin structure at different time intervals are also presented. (**c**) Intermolecular H-bond interactions of ERp57 with tapasin over 1000 ns of MD simulations. In the diagram, interacting pairs of residues are coloured according to their H-bond occupancy over 1000 ns.

The generated root-mean-square fluctuation (RMSF) plot based on the Cα atoms of ERp57 in the apo state and in complex with tapasin, suggests that high flexibility was observed for residues that are responsible for the complex formation with tapasin (Figure 3). Moreover, the residue fluctuations for a and a’ domain were higher for apo ERp57 structure compared to ERp57-tapasin complex. Thus, the increased fluctuations observed for a and a’ domains of wild-type ERp57 are attributed to the domain rearrangements and formation of two distinct “open” and “closed” conformations as shown in Figure 3. The hydrogen bond (H-bond) interactions formed between a and a’ domains of wild-type ERp57 in apo state resulted in a stable closed conformation (Figure 4a). Residues Asn93, Ser98, Gly99 from a domain and Glu368, Glu372 from a’ domain in ERp57 formed dominant and stable intramolecular contacts with ≥ 10% H-bond occupancy (Figure 4a).

These findings from the MD simulations of ERp57-tapasin complexes from our analysis complement the work of Kozlov et al., 2006 [45] by describing that the relative orientation and spacing of a and a’ domains of ERp57 are fixed by their interaction with tapasin. Moreover, the MD simulations can capture the atomic resolution behaviour of biological systems that covers a spatiotemporal domain, where experimental characterization may be difficult [46]. The X-ray models by Kozlov et al., 2006 [45] showed that ERp57 adopts a U shape in solution in the absence of tapasin, and also suggested that the flexibility between domains in both tapasin and ERp57 appears to be limited. Whereas, in our MD analysis the structure of ERp57 formed closed conformation in the absence of tapasin through H-bonds between a and a’ domains, and the C-terminal region of tapasin was also found to be flexible during the MD simulations.

For the tapasin protein the RMSDs were higher by ~4 Å in the apo-form compared to that bound with ERp57 (Figure 4b). The RMSD values for apo tapasin were increased from 5 Å to 12 Å over the second half of the MD simulation, and were mainly due to the motions of C-terminal domain towards the β-b* region (Figure 4b and Figure S3). Higher flexibility of the C-terminal region of tapasin can be explained by the tapasin-MHC I model described by Fisette et al. [47], showing that the C-terminal transmembrane anchor domains of tapasin contacts the CD8 loop (α3 domain) of MHC-I. For the tapasin structure in complex with ERp57, RMSDs were found to be almost stable with ~5 Å throughout the MD simulation. Despite the C-terminal domain of tapasin lacking any involvement in ERp57 binding, this region was found to be quite stable in ERp57-tapasin complex compared to simulated apo-form of tapasin (Figure 4b and Figure S3).

The average number of intermolecular H-bonds formed between ERp57 and tapasin was ~4 during the first 600 ns of MD simulation, which then increased upto ~8 H-bonds during the last 400 ns. Inter-protein H-bonds between ERp57-tapasin occurring for longer than 10% of the simulation were recorded (Figure 4c). The findings suggest that seven intermolecular interactions were found to be stable with occupancy ≥ 20% and the interacting pairs (ERp57/tapasin) of residues were: Gly407/Ala217, Tyr100/Cys95, Tyr100/Asp100, Trp405/Ala217, Ser98/Arg97, Gly449/Gly203, and Val447/Asn205, respectively (Figure 4c). Visualizing the ERp57-tapasin complex behaviour and their protein–protein interacting residues (Figure 4c), suggests that the high number of H-bonds during the end of the MD simulations is due to the increased closeness between both proteins (Figure 3a and Figure S4). These ERp57 and tapasin proteins were observed to make movements in the direction of their respective partner over the time course of the MD simulation (Figure S4).

The wild-type ERp57-tapasin complex was simulated for 1000 ns in two different forms, i.e., the ERp57 and tapasin proteins bonded through disulphide covalent bond Cys57(ERp57)-Cys95(tapasin) and in the non-bonded form (Figure S2a). Analysing the structural stability and flexibility of wild-type ERp57 and tapasin, it was observed that both forms (bonded or non-bonded) have shown similar trend in RMSD and RMSF fluctuations (Figure S2). However, the number of H-bond interactions formed between ERp57-tapasin differs in bonded and non-bonded form during initial 700 ns of MD simulation, which were almost similar (~8 H-bonds) during the end of the MD simulations (Figure S2d). During initial 700 ns, the bonded ERp57-tapasin formed about 4–8 H-bonds, whereas in the non-bonded form ERp57 and tapasin made ~6–10 H-bonds interactions with each other (Figure S2d). In addition, it has been observed from the intermolecular interactions that in the non-bonded form more residues have binding properties with low occupancy (%) to form ERp57-tapasin complex, compared to that of the bonded-form (Figure S2d). These findings suggest that the formation of a disulphide bond between Cys57(ERp57)-Cys95(tapasin) reduces low occupancy intermolecular H-bond interactions, forming a slightly more stable and rigid system. Apart from forming a covalent disulphide bond, residues Cys57(ERp57) and Cys95(tapasin) in the bonded-form were also involved in making stable H-bond interactions with other residues of the complex, whereas in the non-bonded form only the Cys95 residue from tapasin formed such H-bond interactions (Figure S2d).

### 2.2. Insights into the Effects of Cancer Variants at the ERp57-Tapasin Interface

To understand the mutational effects on the ERp57-tapasin complex, two single amino acid mutations; one from ERp57(H408R) and the second from tapasin(P96L) were studied by MD simulations (100 ns) with respect to the wild-type. H408R(ERp57) was placed in the vicinity of the intramolecular disulphide bond (Cys405-Cys409), and P96L(tapasin) was located in the region of the intermolecular disulphide bond between Cys57(ERp57)-Cys95(tapasin) residues. These two mutants were studied in its monomer form as well as when they were in the ERp57-tapasin complex. In order to make comparative analyses with wild-type and mutant systems, we extracted the first 100 ns trajectory from wild-type MD simulations of 1000 ns, and used the extracted trajectory as a representative of the wild-type system. Since the RMSDs (ERp57+tapasin) of the ERp57-tapasin wild-type complex suggests that the average RMSD for 1–100 ns (~8.50 Å) was almost similar to the final frames 900–1000 ns of the MD production run (~9.15 Å) (Figure S5). Furthermore, residues that were involved in forming H-Bond between the ERp57 and tapasin proteins in the complex with occupancy ≥ 20% in 1–1000 ns were all found to be common during the 1–100 ns, for the respective ERp57 or tapasin protein (Figure S5).

**Figure 5:**
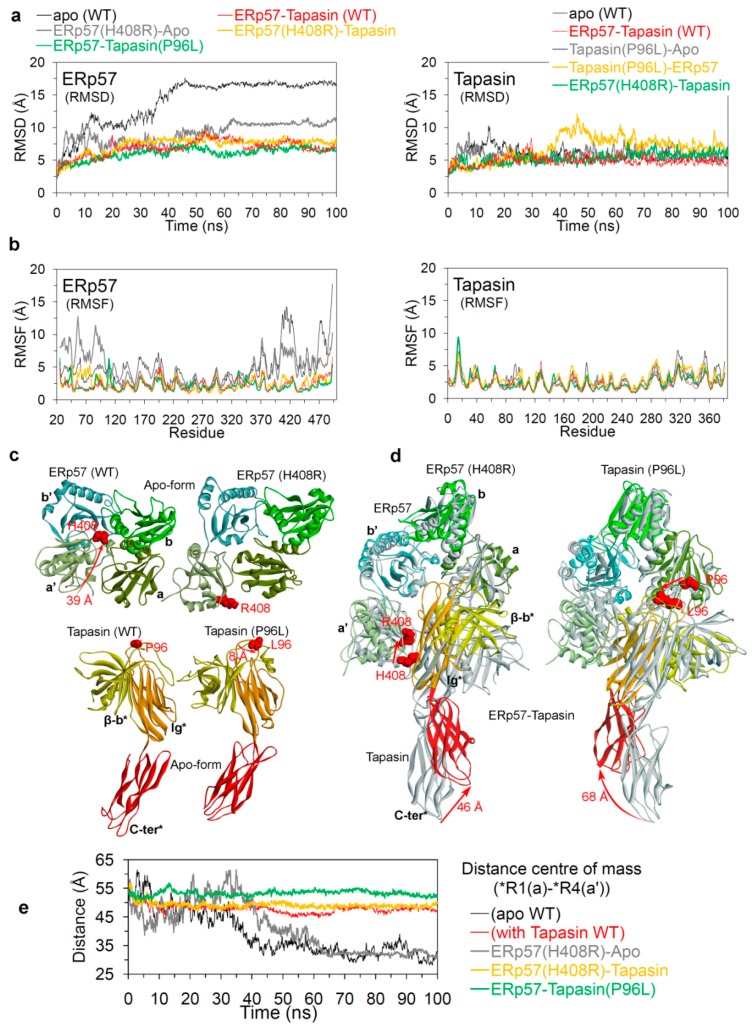
Structural analysis of mutant ERp57(H408R) and tapasin(P96L) systems in comparison with the wild-type systems. (**a**) RMSD of all-atoms (excluding hydrogen atoms) of ERp57 and tapasin from wild-type and mutant systems. (**b**) The residual fluctuations (RMSF) of each residue of ERp57 and tapasin in simulated wild-type and mutant systems. (**c** and **d**) Structure conformations or domain motions of ERp57 and tapasin structures in different simulated systems. (**e**) Distance of mass centre between a and a’ domains in ERp57 structure plotted as a function of time.

An analysis of ERp57 in complex with tapasin, suggests that the structure of ERp57 has obtained RMSDs of ~5–7 Å and the RMSD values remained almost stable throughout the MD simulation (Figure 5a). In the case of the ERp57 apo-form systems, continuous jumps were observed in the RMSDs values during initial 60 ns, particularly, the wild-type ERp57 stabilised at ~17–18 Å. In addition, the mutated apo-form of ERp57(H408R) has obtained relatively less RMSDs of ~10 Å (Figure 5a). The findings from RMSFs suggest that ERp57 in complex with tapasin has obtained similar trends in conformational flexibilities which reached approximately upto 5 Å (Figure 5b). However, the terminal regions/domains (a and a’) have obtained higher RMSFs for ERp57 apo structure, whereas the binding of tapasin with a and a’ domains of ERp57 reduced the flexibility of these regions in ERp57-tapasin systems (Figure 5b). Looking at the tapasin perspective, the RMSDs remained relatively stable with ~5–8 Å throughout the MD, suggesting that presence/absence of ERp57 in the system does not affect the tapasin structure. Similarly, a metric of residue flexibility (RMSF) for tapasin has also shown similar distributions in all systems with RMSFs of ~5 Å (Figure 5b).

The a and a’ domains of ERp57, and the C-terminal region of tapasin were found to adopt different conformations in apo-form and in ERp57-tapasin complex (Figure 5c,d). Secondly, the analysis of the distance centre of mass between the a and a’ domains in the apo-ERp57 and ERp57-tapasin systems also support these conformation changes (Figure 5e). In the case of all three simulated ERp57-tapasin complexes, the a and a’ domain distance centre of masses were found to be stable in ranges of ~50–55 Å, whereas for the apo ERp57 systems, there were jumps in the distances during initial 50 ns of simulation (Figure 5e). Sequentially, during the last 50 ns of simulation time, the apo ERp57 systems obtained relatively short distance centres of mass of ~30 Å and such short distances suggest the association of a and a’ domains to form closed conformation of apo ERp57 (Figure 5). Comparing the ERp57-tapasin structures extracted from the beginning and end of P96L and H408R MD simulations, it was observed that the C-terminus of tapasin moved in the opposite direction from its native structure in both systems (Figure 5d). Particularly, for the P96L(tapasin) mutant system, the C-terminus domain from tapasin move to a distance of 68 Å by the end of MD from its native structure, and for the H408R(ERp57) system it moved to a distance of 46 Å (Figure 5d).

#### Hydrogen Bond Interactions and the Conformation Dynamics

**Figure 6:**
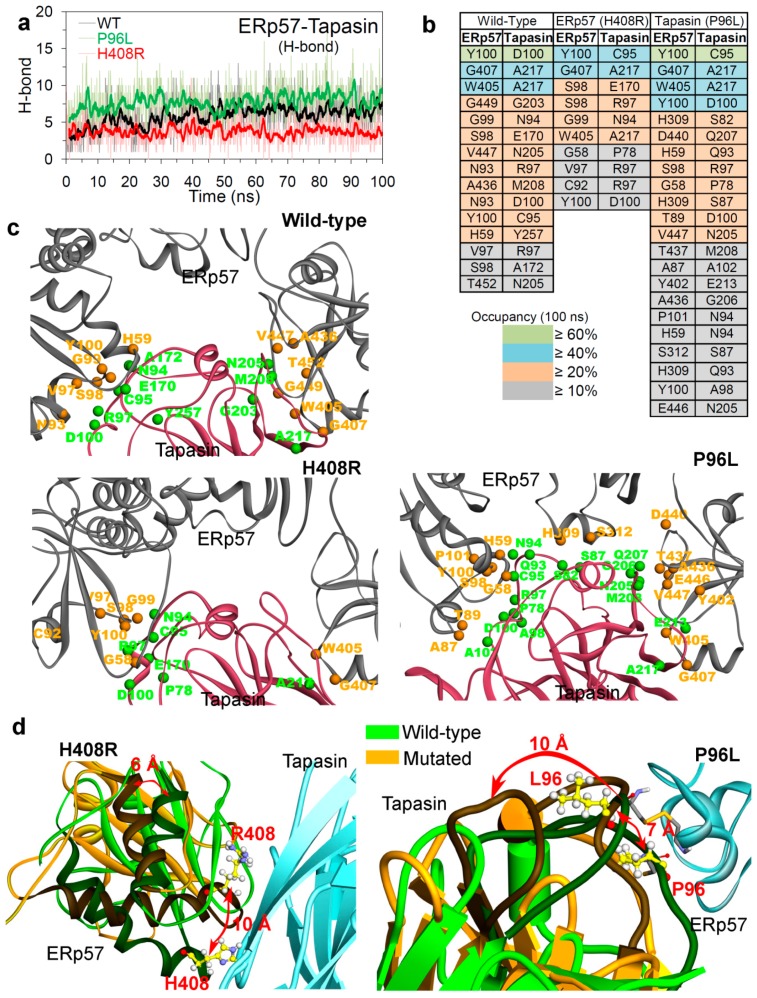
Intermolecular hydrogen bonds formed in the ERp57-tapasin complexes. (**a**) Number of H-bonds formed between ERp57 and tapasin over 100 ns of MD simulations. (**b** and **c**) Functional residues of ERp57-tapasin involved in H-bond formation. In the diagram, interacting pairs of residues are coloured according to their H-bond occupancy over 100 ns of MD simulation. (**d**) Inward conformation change of mutation H408R (reduces ERp57-tapasin interactions) and outward conformation of P96L mutant (increases ERp57-tapasin interactions). Colour scheme: carbon in yellow, oxygen in red, hydrogen in silver, nitrogen in blue, and sulphur in orange.

The effects of cancer mutations on the protein–protein (ERp57-tapasin) interactions were analysed during the MD simulations (Figure 6a), and it was observed that the P96L mutation in tapasin improved the binding of ERp57-tapasin compared to that of the H408R(ERp57) mutant. In addition, the average number of interactions between ERp57-tapasin in wild-type, P96L, and H408R systems were: ~6, ~8, and ~4, respectively (Figure 6a). Additionally, comparing the protein–protein intermolecular interactions with the distance between a-a’ domain of ERp57 in mutated P96L system (Figure 5e), suggests that the high distance (open conformation) was due to stable interactions between ERp57-tapasin (Figure 6a). Analysing the Cys57(ERp57)-Cys95(tapasin) disulphide bonded and non-bonded systems (Table S1 and Figure S6), it was observed that P96L mutation has obtained more interaction (average ~8 H-bonds) in the bonded form compared to the non-bonded form (average ~4 H-bonds). Whereas, the H408R mutant system lacks any such significance difference in the Cys57(ERp57)-Cys95(tapasin) disulphide bonded and non-bonded systems (Figure S6).

Comparing the average number of H-bonds formed over time between ERp57-tapasin (Figure 6a), it was observed that more residues were involved in the intermolecular interactions from the P96L mutant system (Figure 6b) compared to that in the wild-type and H408R mutant systems. Tracing residues forming the interacting pocket or the network at the ERp57 and tapasin interface, suggests that residues Val97, Ser98, Tyr100, Trp405, Gly407 in ERp57 and Asn94, Cys95, Arg97, Asp100 in tapasin formed common H-bond interactions in wild-type, P96L, and H408R systems (Figure 6b,c). In addition, the residues pair (ERp57-tapasin) Tyr100-Asp100, Gly407-Ala217, Trp405-Ala217, and Tyr100-Cys95 were found common forming intermolecular interactions in all three simulated systems (Figure 6b). Residue Gly58 in ERp57 and Pro78 in tapasin were common residues involved in H-bond interactions only in the mutated systems (P96L and H408R). Furthermore, ERp57 residues that were common in wild-type and P96L/H408R were His59, Ala436, and Val447/Gly99, respectively (Figure 6b,c), and the tapasin residues that were common in wild-type and P96L/H408R were Asn205, Met208, and Ala217/Glu170, respectively (Figure 6b,c).

Visualizing and tracing the movement of residues in the ERp57-tapasin binding interface suggest that during the MD simulation of H408R(ERp57) the mutated system obtained an inward interface, whereas the mutated system P96L(tapasin) opened outwards (Figure 6d). This finding correlates with the H-bond interaction analysis that the P96L mutated system has obtained a high number of H-bonds (as tapasin opens outwards and increases binding interface) interactions compared to that of the H408R system (Figure 6a,d). Particularly, for the mutation H408R system the movement observed was about 10 Å that shifted from its native position to an inward conformation of the ERp57-tapasin binding interface. In addition, the mutation P96L system shifted about 7 Å outward in the ERp57-tapasin binding interface which caused a nearby loop residue range: 164-184 moved ~10 Å from its native position (Figure 6d). These findings about the dynamic behaviour of the ERp57-tapasin complex and intermolecular H-bond interactions observed during the MD simulations (Figure 6), brings a more detailed picture for this protein–protein (ERp57-tapasin) complex compared to that of initial (ΔΔG or Δstability) structural stability change (Figure 2). Since in the structure stability change (ΔΔG) prediction upon mutation (Figure 2) only the active zone (i.e., residue up to 4.5 Å from a residue to be mutated) is considered as flexible and the rest of the protein is in the rigid state, whereas in the MD simulation the entire ERp57-tapasin complex is in a flexible environment surrounded by solvents. In addition, this flexibility allows the rearrangements in the ERp57-tapsin complex to form stable H-bond bindings (Figure 6).

**Figure 7:**
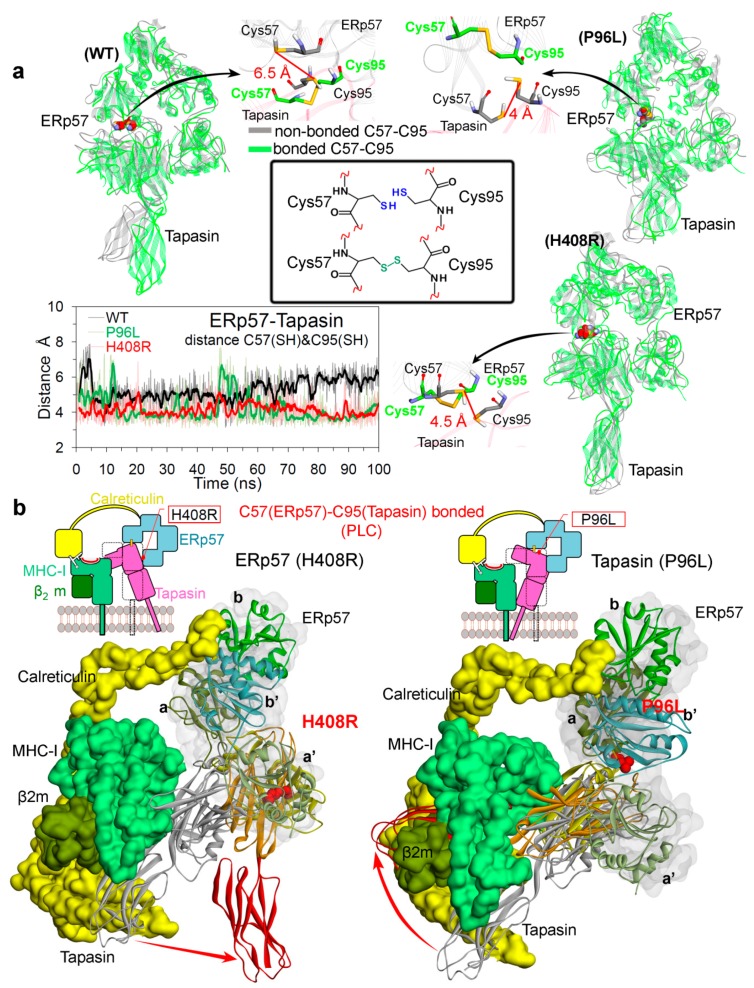
Conformation dynamics of the ERp57-tapasin complex. (**a**) Distance between Cys57(ERp57)-Cys95(tapasin) in the absence of disulphide bond, and conformations of residues and ERp57-tapasin complex compared with disulphide bonded and non-bonded form (coordinates extracted from the end of the MD simulations). (**b**) Structure extracted from the end of the MD simulation and compared with the PLC complex (PDB: 6ENY [4]). ERp57 or tapasin coloured in grey are wild-type from PDB: 6ENY [4], and with their domains coloured are from mutated systems (extracted from the MD simulations). Colour scheme: oxygen in red, hydrogen in silver, nitrogen in blue, and sulphur in orange.

In vivo evidences suggest that covalent ERp57-tapasin heterodimer is the functional unit responsible for the peptide editing [5,27,28], and is 50–85% covalently bonded in cells activated with interferon-γ [23,24,25,26]. Therefore, to understand the dynamics of the disulphide bond between Cys57(ERp57)-Cys95(tapasin) on the ERp57-tapasin complex, systems were also simulated in the absence of a disulphide bond between two cysteines (Cys57 and Cys95; Figure 7a). Coordinates of ERp57-tapasin extracted from MD were superimposed with each other, and it was observed that for ERp57 overall there was a similar trend of conformational changes in both forms (bonded/non-bonded) for the wild-type, P96L and H408R systems (Figure 7a). However, the C-terminal domain of tapasin in the wild-type system has slightly more movement in the non-bonded form compared to the bonded form. In addition, for the P96L mutant system in both forms, the ERp57 and tapasin had almost similar conformational change, except for some residues (ranging 305–317) in ERp57 have shown movements towards the tapasin protein in the bonded form (Figure 7a). Moreover, analysing the distance between SH (sulphur) atoms of two Cys57(ERp57) and Cys95(tapasin) in non-bonded forms, it was observed that the wild-type system has more distance compared to the other two (P96L and H408R) mutant systems (Figure 7a).

To postulate the conformational change with respect to the native starting structure, the simulated ERp57-tapasin mutant models were superimposed with the PLC complex (PDB ID: 6ENY [4]), and it was observed that C-terminus of tapasin have some significant movements upon mutation (Figure 7b). The mutations H408R(ERp57) and P96L(tapasin) located at the ERp57-tapasin interacting interface have different effects on the C-terminus of tapasin. For mutant H408R in ERp57, the C-terminus of tapasin moved away from the PLC and in the P96L system it showed movements towards the PLC (Figure 7b). Particularly, the C-terminus domain of tapasin is involved in the interactions with the CD8 loop (α3 domain) of MHC-I molecules from the peptide-loading complex [47]. Therefore, it can be suggested that these movements of C-terminus from the tapasin protein can have a significant effect on the peptide presentation. In addition, these findings suggest that mutations at precise positions at the ERp57-tapasin (protein–protein) interface may have an impact on antigen presentation in tumour cells, allowing them to escape from recognition by the human immune system.

## 3. Material and Methods

### 3.1. Protein Preparation

ERp57-tapasin thiol oxidoreductase heterodimer coordinates were taken from an X-ray crystal structure retrieved from the Protein Data Bank (http://www.rcsb.org/pdb; PDB ID: 3F8U) [22,48]. The missing residue coordinates in the tapasin structure (residues range: 12–19, 28–33, and 171–176) were built using the “Prepare Protein” protocol of BIOVIA Discovery Studio Client v18.1 program (Dassault Systemes, BIOVIA Corp., San Diego, CA, USA) (Figure 1b). The parameters in this protocol were set as; CHARMM (Chemistry at HARvard Macromolecular Mechanics) forcefield, pH 7.4, protein dielectric constant equal to 10, and ionic strength equal to 0.145 M. In addition, other parameters in this protocol were set as default values. The resulting full protein structures were energy minimised by a “smart minimiser” algorithm in the BIOVIA Discovery Studio Client v18.1 program. Moreover, during minimization the non-bonded list radius was set to 14 and the root-mean-square (RMS) gradient was set to 0.1. The optimised structures were further used for mutation analysis and molecular dynamic simulations.

### 3.2. In Silico Mutation and Molecular Dynamics Simulation

The structural perturbations caused by each mutation (∆stability or ∆∆G) were calculated by the “Residue Scan” module of the Molecular Operating Environment (MOE; Chemical Computing Group Inc.) package. In this method, each of the selected interface residues can be replaced with a specific variant of interest, and the effect of the mutation on the structural stability change (∆stability or ∆∆G) or the binding free energy (∆G_bind_) of the complex can be computed. In this work the LowModeMD ensemble was used to compute the change in the structural stability (∆stability or ∆∆G) upon mutation. In this approach residues farther than 4.5 Å distance (from a residue to be mutated) are considered fixed or rigid, and residues within this (4.5 Å) distance are flexible (i.e., the active zone). Parameters used for the structural stability change calculations involve; CHARMM27 forcefield, LowModeMD ensemble, and 10,000 search iterations. The ∆stability (kcal/mol) values were calculated as the relative binding free energy difference (∆∆G_bind_) between the mutant (∆G_mutant_) and wild-type (∆G_wild-type_). A negative ΔΔG represents a more stable complex (ΔGmut>ΔGwild) and a positive ΔΔG represents a destabilised complex (ΔGmut<ΔGwild) [35,49].

GROMACS 4.6.5 [50,51] program (GROMACS; Groningen Machine for Chemical Simulations) was used to perform molecular dynamic calculations for all 10 modelled systems assigning the Gromos96 43a1 forcefield (Gromos; GROningen Molecular Simulation) [52]. Four wild-type systems on which 1000 ns molecular dynamics simulations performed were: (i) ERp57 (monomer), (ii) tapasin (monomer), (iii) ERp57-tapasin (Cys57-Cys95 disulphide bonded), and (iv) ERp57-Tapasin (Cys57-Cys95 non-bonded). The six mutated systems on which 100 ns of the MD simulations performed were: (i) ERp57 (H408R; monomer), (ii) tapasin (P96L; monomer), (iii and iv) ERp57-tapasin (H408R ERp57 mutated) Cys57-Cys95 disulphide bonded and non-bonded, and (v and vi) ERp57-tapasin (P96L tapasin mutated) Cys57-Cys95 disulphide bonded and non-bonded.

For the simulation box, the periodic boundary conditions were applied in all directions to avoid finite size effects, and the appropriate number of Na^+^ and Cl^-^ ions were added to neutralise overall charge. The water molecules were modelled by simple point charge (SPC) parameters. To remove bad contacts from the initial structure, the systems were then relaxed for 20,000 steps of the steepest-descent energy minimization process, or until the minimum energy of the system was obtained. Following energy minimization, to allow adjustment of solvent molecules with counter ions the equilibration of the systems was carried out for 1000 ps (1 ns) using the NPT (number of particles (N), system pressure (P), and temperature (T); isobaric-isothermal) thermodynamic ensemble. The long-range electrostatics were calculated using the particle mesh ewald (PME) method [53], and the LINCS (LINear Constraint Solver) constraint or algorithm [54] was used to restrain the bond lengths (between the heavy atoms and the complementary nonpolar hydrogen atoms). A cutoff of 10 Å was set for van der Waals and Coulomb interactions. The temperature was maintained constantly at 300 K by a velocity-rescaling thermostat [55], and for the constant 1.0 bar pressure the Parrinello–Rahman barostat [56] was used. Properties such as the density, pressure, and temperature were traced for each system (Figure S7) after the equilibration process, and it was observed that they remained stable, representing an equilibrated system. These equilibrated systems were subsequently used to perform final MD production runs; the wild-type systems were simulated for 1000 ns and systems with mutations were simulated for 100 ns using a leapfrog integrator [57]. The leapfrog integrator is an algorithm implemented in the GROMACS program, to solve the Newton’s equations of motion by integration. In the leapfrog algorithm, position (r) and velocities (v) are leaping like frogs over each other’s backs. Subsequently, the coordinates obtained from the molecular dynamics calculations were saved every 10 ps of time in the trajectories that were used further for analysis.

The results from the production run or the trajectories from MD simulations were further analysed using GROMACS [50,51], BIOVIA Discovery Studio [Dassault Systemes, BIOVIA Corp., San Diego, CA, USA], and visual molecular dynamics (VMD) tools [58]. Along with the libraries for the MD simulations, the GROMACS package provides several modules for the analysis of the trajectories obtained as an output from MD. The GROMACS modules were used to trace change in the temperature, pressure, density, different types of energies (e.g., potential), RMSDs, and for H-bond analysis. The BIOVIA Discovery Studio program has an interface that involves tools for modifying and viewing data, running protocols, and analysing the results. The “superimpose proteins” module was used to superimpose the xyz coordinates of ERp57 or tapasin protein retrieved from the MD simulations. VMD is a molecular visualiser and analysis program designed for several biological systems (protein, nucleic acids, etc.), and supports a large number of molecular dynamics simulation packages. The VMD software was used in this work to analyse and visualise the dynamic behaviour of proteins obtained from the MD simulations, and analysis such as RMSD, RMSF, H-bond, and conformational change (by extracting xyz coordinates from MD) was traced using this program. The RMSDs of the ERp57 and tapasin proteins were computed indicating a change from the initial structure by two-step process. At first the RMS alignment was performed that fits molecules considering the select groups of atoms (e.g., protein), and moves molecules to new positions from the MD trajectory. As the following step, the RMS distance (RMSD) between two molecules (frames) from MD was computed. The RMSD was computed for all atoms, excluding the hydrogens of ERp57 or tapasin protein. RMSF were calculated based on the Cα atoms, and the H-bonds were computed considering the donor–acceptor distance cutoff ≤ 3.5 Å and donor-H-acceptor angle cutoff ≥ 160°−180°.

## 4. Conclusions

The effects of cancer-related variants in the ERp57 and tapasin proteins at the protein–protein (ERp57-tapasin) interface were studied using different computational approaches. The initial in silico mutagenesis analysis identified that H408R(ERp57) and P96L, D100A, G183R(tapasin) improved the structural stability (ΔΔG) of respective proteins. Particularly, the single amino acid variant H408R from the catalytic site placed in the vicinity of intramolecular disulphide bond (Cys405-Cys409) in ERp57, and the variant P96L in the region of intermolecular disulphide bond between Cys57(ERp57)-Cys95(tapasin) were proposed to have a significant impact on the protein–protein binding. Therefore, MD simulations were performed for the wild-type and mutant (P96L and H408R) systems, as well as for systems where Cys57(ERp57)-Cys95(tapasin) were in the non-bonded form.

The H-bond network analysis suggested that the P96L(tapasin) mutant improved ERp57-tapasin binding compared to that of mutant H408R(ERp57) and the wild-type system (average number of ERp57-tapasin interaction was: ~6(wild-type), ~8(P96L), and ~4(H408R)). The dynamics and conformational changes revealed that the H408R(ERp57) mutant system obtained an inward ERp57-tapasin interface, whereas in the P96L(tapasin) mutant system this interface opened outward increasing the ERp57-tapasin interactions.

Residues involved with high occupancy (%) protein–protein interactions were identified during the MD simulations, representing stable ERp57-tapasin bindings. The common residues in the wild-type and mutant (P96L and H408R) systems were: Val97, Ser98, Tyr100, Trp405, Gly407 in ERp57 and Asn94, Cys95, Arg97, Asp100 in tapasin. In addition, the residue pairs (ERp57-tapasin) Tyr100-Asp100, Gly407-Ala217, Trp405-Ala217, and Tyr100-Cys95 were found to be common in all (wild-type, P96L, and H408R) simulated ERp57-tapasin complexes.

For the ERp57 protein, open and closed conformations were observed in the presence and absence of tapasin, and this was supported by the distance centre of masses between a-a’ domains of ERp57 (distance in WT (wild-type) apo-form was ~28 Å and in the presence of tapasin was ~45 Å). In the Cys57(ERp57)-Cys95(tapasin) disulphide bonded and non-bonded systems a significant difference was observed in the P96L(tapasin) mutant, where a higher number of ERp57-tapasin interactions (~8 H-bonds) was observed in the bonded form compared to the non-bonded state (~4 H-bonds).

The simulated ERp57-tapasin mutant models superimposed with the native peptide-loading complex, suggest that in both mutant systems the C-terminus domain in tapasin displaced in the opposite direction with respect to the native structure. For mutant H408R(ERp57) the C-terminus domain shifted away from the PLC, whereas in the P96L system it shifted towards the PLC (this C-terminus domain is involved in binding with the MHC-I molecule). Therefore, it can be proposed that mutants at the precise positions in the ERp57-tapasin (protein–protein) interface may have a significant effect on antigen presentation in tumour cells, however, the actual effect and mechanisms can be studied by in vitro experimental techniques or by simulating the whole peptide-loading complex. Findings from this study can help to understand the structural properties of two important factors (ERp57 and tapasin) from the MHC-I pathway, and can have implications for the function of the PLC and, ultimately, for the presentation of MHC-I peptide complex.

## Supplementary Materials

The following are available online at https://www.mdpi.com/2072-6694/12/3/737/s1, Figure S1: The PDIA3(ERp57) and TAPBP(tapasin) genes altered in different cancer types, Figure S2: Cys57-Cys95 disulphide bonded and non-bonded forms in the wild-type systems, Figure S3: RMSF of the wild-type tapasin, Figure S4: Structural change in the ERp57-tapasin (wild-type system), and protein–protein interactions from MD, Figure S5: Extracted 100 ns trajectory from the wild-type MD simulations of 1000 ns, and used it as a representative to compare results with mutant systems (100 ns), Figure S6: H-bond interactions from different disulphide bonded/non-bonded systems, Figure S7: Properties of the equilibrated (1000 ps; 1 ns) wild-type and mutated systems using NPT ensemble (constant number of particles, pressure, and temperature), Table S1: Residues involved in ERp57-tapasin binding (Cys57-Cys95 bonded/non-bonded).
