# Supplementary material for: Recognition Dynamics of Cancer Mutations on the ERp57-Tapasin Interface
Source: Cancers (Basel). 2020 Mar 20;12(3):737. doi: 10.3390/cancers12030737 (PMC7140079; doi:10.3390/cancers12030737)
Supplement: Supplementary file 1 [file cancers-12-00737-s001.pdf]

Supplementary Materials: Recognition Dynamics of Cancer Mutations on the ERp57-Tapasin Interface

Monikaben Padariya, Umesh Kalathiya, Douglas R. Houston and Javier Antonio Alfaro

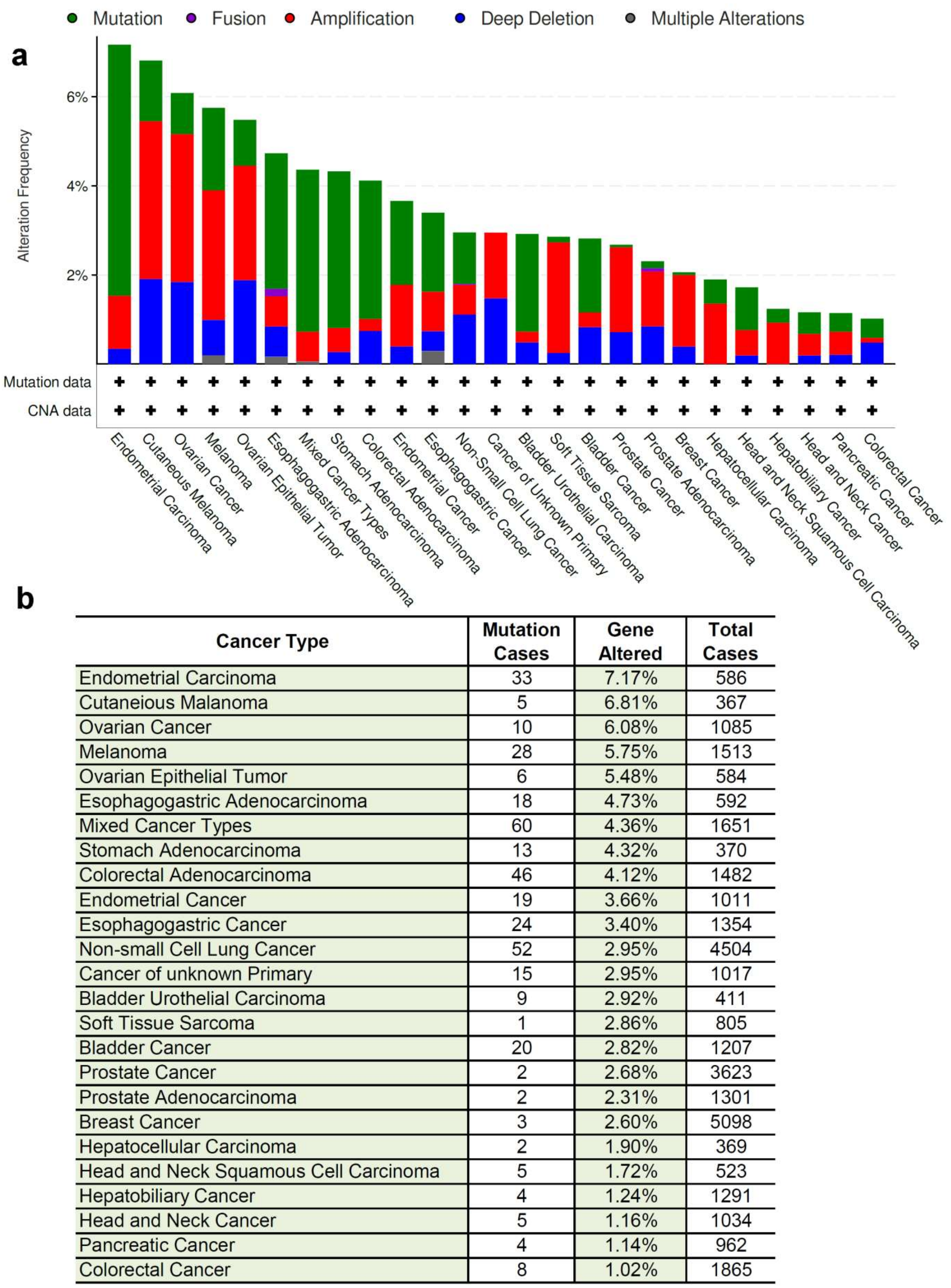

b

Cancer Type	Mutation Cases	Gene Altered	Total Cases
Endometrial Carcinoma	33	7.17%	586
Cutaneious Malanoma	5	6.81%	367
Ovarian Cancer	10	6.08%	1085
Melanoma	28	5.75%	1513
Ovarian Epithelial Tumor	6	5.48%	584
Esophagogastric Adenocarcinoma	18	4.73%	592
Mixed Cancer Types	60	4.36%	1651
Stomach Adenocarcinoma	13	4.32%	370
Colorectal Adenocarcinoma	46	4.12%	1482
Endometrial Cancer	19	3.66%	1011
Esophagogastric Cancer	24	3.40%	1354
Non-small Cell Lung Cancer	52	2.95%	4504
Cancer of unknown Primary	15	2.95%	1017
Bladder Urothelial Carcinoma	9	2.92%	411
Soft Tissue Sarcoma	1	2.86%	805
Bladder Cancer	20	2.82%	1207
Prostate Cancer	2	2.68%	3623
Prostate Adenocarcinoma	2	2.31%	1301
Breast Cancer	3	2.60%	5098
Hepatocellular Carcinoma	2	1.90%	369
Head and Neck Squamous Cell Carcinoma	5	1.72%	523
Hepatobiliary Cancer	4	1.24%	1291
Head and Neck Cancer	5	1.16%	1034
Pancreatic Cancer	4	1.14%	962
Colorectal Cancer	8	1.02%	1865

Figure S1. The PDIA3(ERp57) and TAPBP(tapasin) genes altered in different cancer types. (**a** and **b**) Data retrieved from the cBioPortal database (Cancer Discov. 2012, 2, 401-404), and in the cBioPortal the search was limited to 300 total cases and the minimum gene altered in 1%.

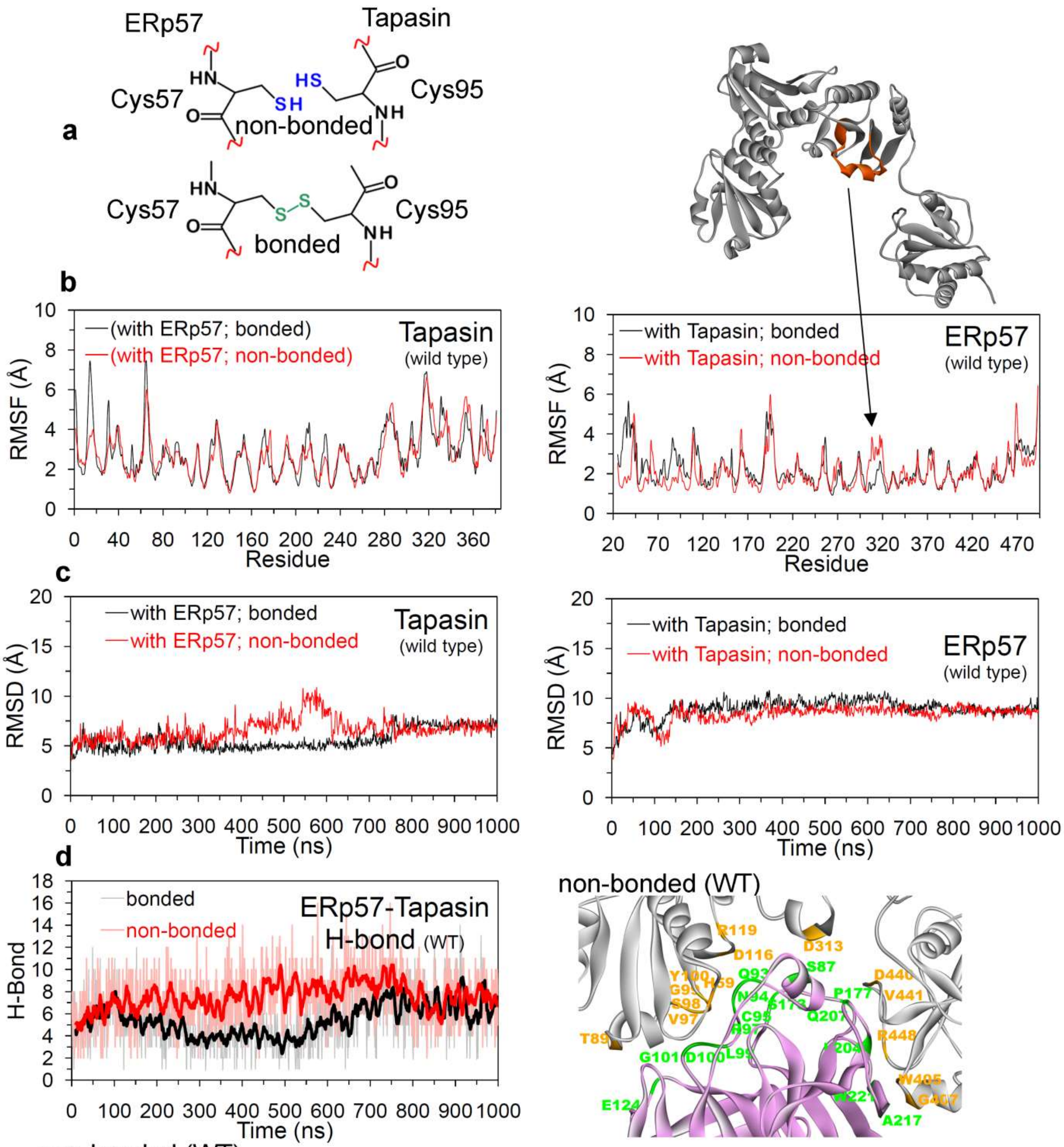

non-bonded (WT)

**ERp57**	G407	S98	W405	D116	G99	D116	Y100	D313	T89	R119	R448	V97	R448	H59	D116	D440	R448	V441	Y100	R119
**Tapasin**	A217	D100	A217	S173	L99	N94	C95	S87	E124	Q93	L204	G101	W221	Q93	R97	Q207	P177	Q207	G101	N94
	≥ 40 %			≥ 30 %			≥ 20 %				≥ 10 %									

bonded (WT)

**ERp57**	G407	Y100	Y100	W405	S98	G449	V447	N90	T89	G58	S169	H59	C57
**Tapasin**	A217	C95	D100	A217	R97	G203	N205	E170	E124	Y257	N93	N94	Y257
	≥ 40 %			≥ 30 %	≥ 20 %			≥ 10 %					

Figure S2. Cys57-Cys95 disulphide bonded and non-bonded forms in the wild-type systems. (**a**) Cys57(ERp57)-Cys95(tapasin) in presence/absence of disulphide bond. (**b**) The residual fluctuations (RMSF) of each residue of ERp57 and tapasin in simulated disulphide bonded and non-bonded systems. (**c**) RMSD of all-atoms (excluding hydrogen atoms) of ERp57 and tapasin from simulated disulphide bonded and non-bonded systems. (**d**) Intermolecular interactions observed between ERp57-tapasin over 1000 ns of MD simulations. In the diagram, residues (interacting pairs) are colored according to their H-bonding occupancies (over 1000 ns).

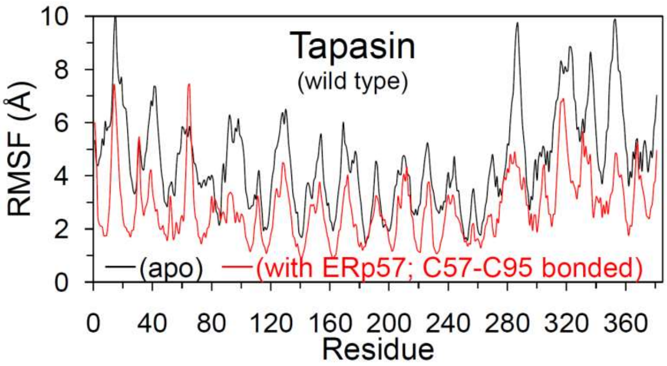

Figure S3. RMSF of the wild-type tapasin. RMSF plot based on the Cα atoms of tapasin from 1000 ns MD simulations for the apo-form of tapasin, and when complexed with ERp57.

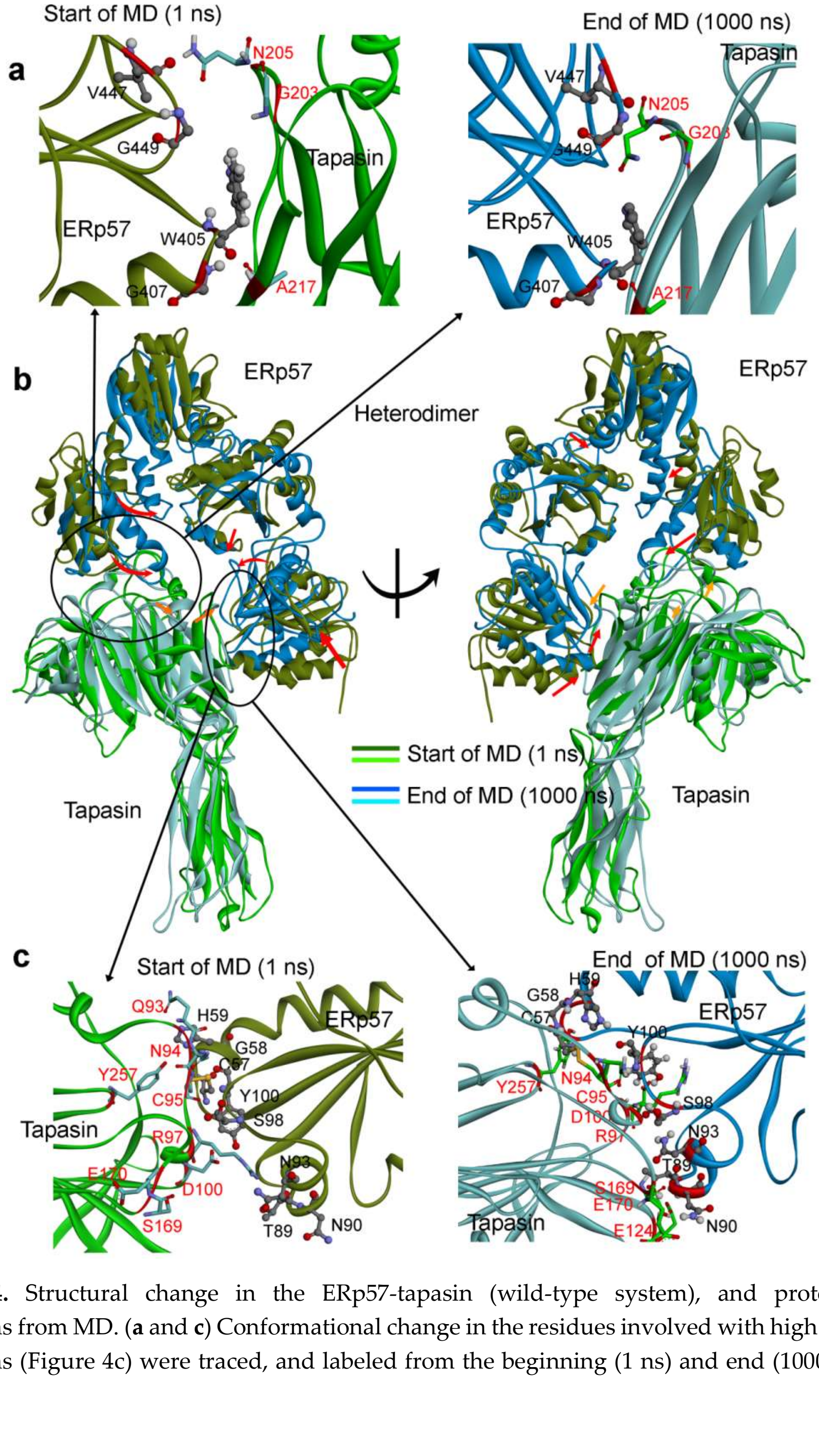

Figure S4. Structural change in the ERp57-tapasin (wild-type system), and protein-protein interactions from MD. (**a** and **c**) Conformational change in the residues involved with high occupancy interactions (Figure 4c) were traced, and labeled from the beginning (1 ns) and end (1000 ns) of the

MD simulations. (**b**) The structural change of the ERp57-tapasin complex obtained from the beginning (1 ns) and end (1000 ns) of the wild-type MD simulations. Arrow (red in ERp57 and orange in tapasin) represents movement of the ERp57 or tapasin region in pointed direction.

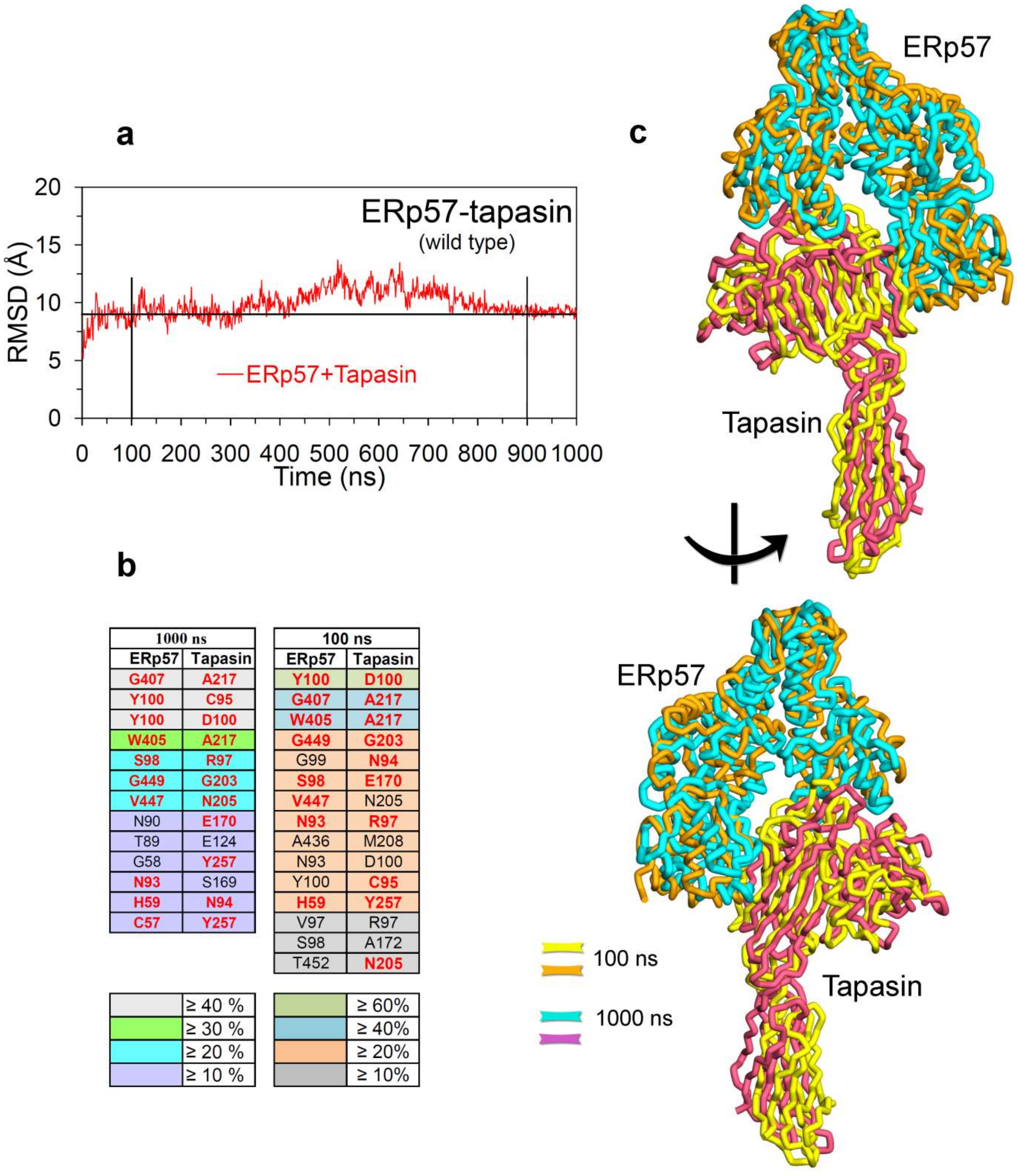

1000 ns	
ERp57	Tapasin
G407	A217
Y100	C95
Y100	D100
W405	A217
S98	R97
G449	G203
V447	N205
N90	E170
T89	E124
G58	Y257
N93	S169
H59	N94
C57	Y257

100 ns	
ERp57	Tapasin
Y100	D100
G407	A217
W405	A217
G449	G203
G99	N94
S98	E170
V447	N205
N93	R97
A436	M208
N93	D100
Y100	C95
H59	Y257
V97	R97
S98	A172
T452	N205

	1000 ns		100 ns
	≥ 40 %		≥ 60%
	≥ 30 %		≥ 40%
	≥ 20 %		≥ 20%
	≥ 10 %		≥ 10%

Figure S5. Extracted 100 ns trajectory from the wild-type MD simulations of 1000 ns, and used it as a representative to compare results with mutant systems (100 ns). (**a**) RMSDs (ERp57+tapasin) of the ERp57-tapasin for the wild-type complex. (**b**) H-bond interactions traced/extracted from the 1–100 ns and the 1–1000 ns simulation of the wild-type system. In the ERp57 protein, except for Gly58, Thr89, and Asn90all other nine residues (Cys57, His59, Asn93, Ser98, Try100, Trp405, Gly407, Val447, and Gly449) were common in 100 ns and 1000 ns analysis. In addition, for the tapasin protein residues Asn94, Cys95, Arg97, Asp100, Glu170, Gly203, Asn205, Ala217, and Tyr257 were present in 100 ns as well as in 1000 ns (except for Glu124 and Ser169). The text in red represents common residues in both 100 ns and 1000 ns, and the background color represents the occupancy of particular H-Bond in 1–100 or 1–1000 ns. (**c**) Superimposed structures of the 100 ns and 1000 ns MD frames, suggest almost similar structural conformation.

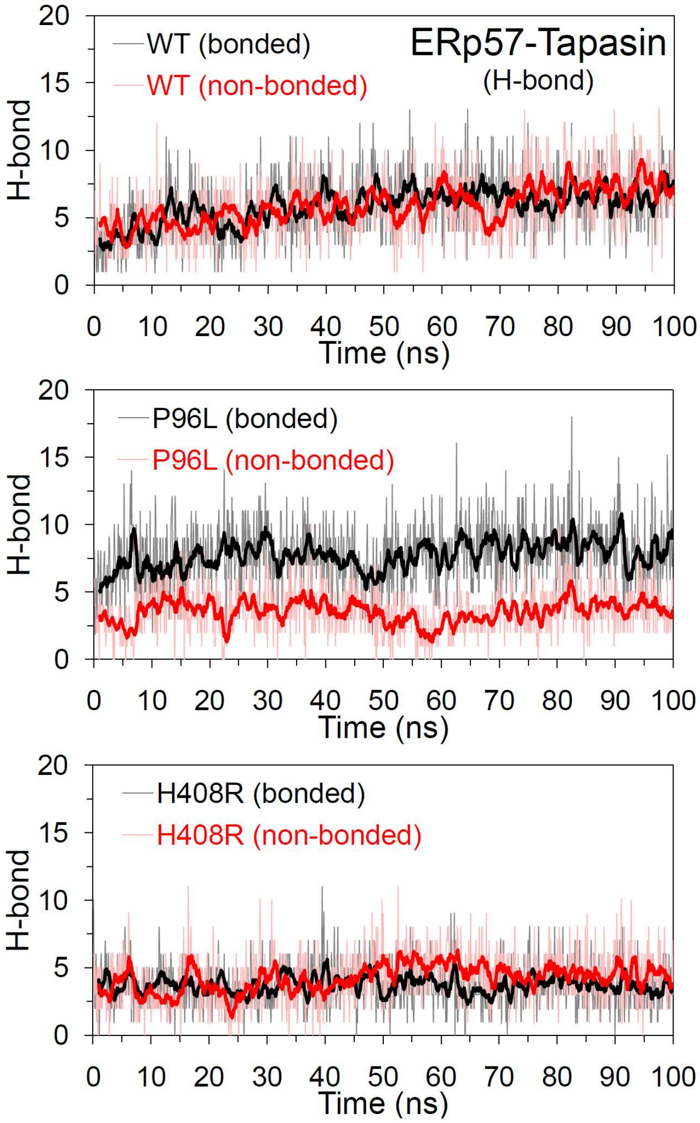

Figure S6. H-bond interactions from different disulphide bonded/non-bonded systems. Intermolecular interactions between ERp57-tapasin in presence/absence of disulphide bond between Cys57(ERp57)-Cys95(tapasin) during 100 ns of MD simulation. The dark lines represent trends with a moving average formed with a period of 1 ns (i.e., number of H-bonds averaged every 1000 ps).

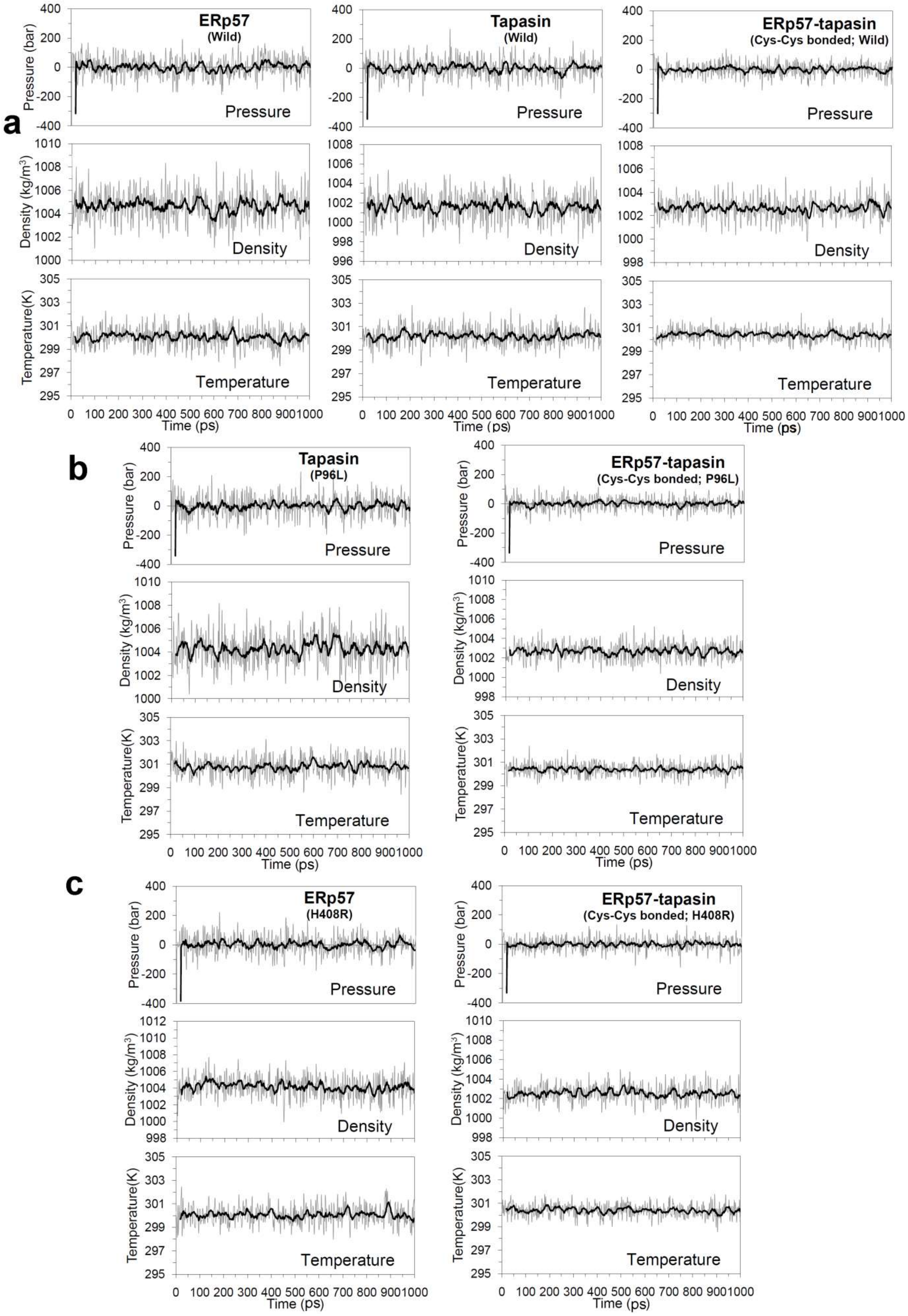

Figure S7. Properties of the equilibrated (1000 ps; 1 ns) wild-type and mutated systems using NPT ensemble (constant number of particles, pressure, and temperature). (**a**, **b**, and **c**) Wild-type, P96L, and H408R systems, respectively. For the ERp57-tapasin system results are presented for the Cys57(ERp57)-Cys95(tapasin) disulphide bonded system. The dark lines represent trends with a moving average with a period of 10 ps (i.e., number of values averaged every 10 ps).

Table S1. Residues involved in ERp57-tapasin binding (Cys57-Cys95 bonded/non-bonded). The intermolecular interactions between ERp57-tapasin in presence/absence of disulphide bond between Cys57-Cys95. Occupancy ≥ 10% (from 100 ns) are presented in this table.

**Wild-Type**			**ERp57-H408R**			**Tapasin-P96L**		
Cys57-Cys95 disulphide bonded (ERp57-tapasin)								
**ERp57**	**Tapasin**	**Occup.%**	**ERp57**	**Tapasin**	**Occup.%**	**ERp57**	**Tapasin**	**Occup.%**
Y100	D100	61.08	Y100	C95	45.11	Y100	C95	75.74
G407	A217	51.20	G407	A217	45.01	G407	A217	44.61
W405	A217	44.31	S98	E170	29.44	W405	A217	43.41
G449	G203	36.33	S98	R97	28.94	Y100	D100	42.61
G99	N94	30.64	G99	N94	26.35	H309	S82	35.93
S98	E170	30.24	W405	A217	22.16	D440	Q207	35.23
V447	N205	27.15	G58	P78	15.67	H59	Q93	32.14
N93	R97	26.75	V97	R97	13.37	S98	R97	30.54
A436	M208	25.15	C92	R97	12.97	G58	P78	29.94
N93	D100	24.75	Y100	D100	10.88	H309	S87	29.64
Y100	C95	22.75				T89	D100	25.25
H59	Y257	22.26				V447	N205	22.65
V97	R97	15.27				T437	M208	16.17
S98	A172	11.08				A87	A102	15.37
T452	N205	10.48				Y402	E213	15.27
						A436	G206	13.97
						P101	N94	12.38
						H59	N94	12.18
						S312	S87	11.68
						H309	Q93	11.48
						Y100	A98	11.18
						E446	N205	10.18
No disulphide bond between Cys57 and Cys95 (ERp57-tapasin)								
G407	A217	42.61	H59	C95	47.31	G58	P78	40.32
W405	A217	38.92	W405	A217	46.31	W405	A217	40.22
R119	Q93	37.62	A217	G407	36.93	Y402	E213	29.44
Y100	C95	31.94	Y100	C95	34.63	R448	N205	22.26
Y100	D100	29.64	R119	N94	22.46	W56	L99	20.66
D440	Q207	27.05	R448	W221	21.36	D440	Q207	18.06
T437	M208	26.15	Y454	D224	18.16	V447	N205	13.77
A438	Q207	23.45	S443	G178	14.37	G407	A217	10.68
R448	W221	21.76	N93	D100	12.48			
S98	D100	17.37	Y100	D100	11.18			
D116	S174	15.07	W405	N205	10.28			
T437	N205	14.97	V97	R97	10.28			
V441	N205	14.17						
V447	G203	13.47						
R62	A92	13.27						
R119	N94	12.67						
G99	L99	11.28						
D116	N94	10.28						

© 2020 by the authors. Licensee MDPI, Basel, Switzerland. This article is an open access article distributed under the terms and conditions of the Creative Commons Attribution (CC BY) license (http://creativecommons.org/licenses/by/4.0/).
